# Effects of Fructans from Mexican Agave in Newborns Fed with Infant Formula: A Randomized Controlled Trial

**DOI:** 10.3390/nu7115442

**Published:** 2015-10-29

**Authors:** Gabriel López-Velázquez, Minerva Parra-Ortiz, Ignacio De la Mora-De la Mora, Itzhel García-Torres, Sergio Enríquez-Flores, Miguel Angel Alcántara-Ortigoza, Ariadna González-del Angel, José Velázquez-Aragón, Rosario Ortiz-Hernández, José Manuel Cruz-Rubio, Pablo Villa-Barragán, Carlos Jiménez-Gutiérrez, Pedro Gutiérrez-Castrellón

**Affiliations:** 1 Laboratorio de Bioquímica-Genética, Instituto Nacional de Pediatría, Mexico City 04530, Mexico; ignaciodelamora@yahoo.com.mx (I.M.-M.); itzheltorres@hotmail.com (I.G.-T); sergioenriquezflores@gmail.com (S.E.-F.); 2 Hospital General de México Dr. Eduardo Liceaga, Mexico City 06726, Mexico; md_parra@hotmail.com; 3 Urgencias Pediatría, Centro Médico ABC, Santa Fe 05300, Mexico; 4 Laboratorio de Biología Molecular, Instituto Nacional de Pediatría, Mexico City 04530, Mexico; malcantaraortigoza@gmail.com (M.A.A.-O.); ariadnagonzalezdelangel@gmail.com (A.G.-A.); jovear2002@gmail.com (J.V.-A.); 5 Departamento de Biología Celular, Facultad de Ciencias, Universidad Nacional Autónoma de México, Mexico City 04510, Mexico; r_oh@ciencias.unam.mx; 6 Nekutli, S.A. de C.V., Zapopan, Jalisco 45070, Mexico; jmcruz1982@gmail.com; 7 Centro Mexicano de Fomento al Desarrollo, Hidalgo 04212, Mexico; pablovillab@yahoo.com.mx; 8 Unidad de Investigación Traslacional & Centro de Análisis de la Evidencia, Hospital General Dr. Manuel Gea González, Mexico City 14080, Mexico; amcrr1962@gmail.com

**Keywords:** soluble fiber, infant microbiota, breast milk, immune response

## Abstract

Background: The importance of prebiotics consumption is increasing all over the world due to their beneficial effects on health. Production of better prebiotics from endemic plants raises possibilities to enhance nutritional effects in vulnerable population groups. Fructans derived from Agave Plant have demonstrated their safety and efficacy as prebiotics in animal models. Recently, the safety in humans of two fructans obtained from *Agave tequilana* (Metlin**^®^** and Metlos**^®^)** was demonstrated. Methods: This study aimed to demonstrate the efficacy as prebiotics of Metlin**^®^** and Metlos**^®^** in newborns of a randomized, double blind, controlled trial with a pilot study design. Biological samples were taken at 20 ± 7 days, and three months of age from healthy babies. Outcomes of efficacy include impact on immune response, serum ferritin, C-reactive protein, bone metabolism, and gut bacteria changes. Results: There were differences statistically significant for the groups of infants fed only with infant formula and with formula enriched with Metlin**^®^** and Metlos**^®^**. Conclusions: Our results support the efficacy of Metlin**^®^** and Metlos**^®^** as prebiotics in humans, and stand the bases to recommend their consumption. Trial Registration: ClinicalTrials.gov, NCT 01251783.

## 1. Introduction

Prebiotics are non-digestible food ingredients that beneficially affect the host by selectively stimulating the growth and/or activity of one or a limited number of bacteria, modulating the composition of the natural ecosystem [1]. They are found in vegetables and fruits and can be industrially processed from renewable materials. Carbohydrates have a positive prebiotic activity score if they are metabolized similarly or as well as glucose by probiotic strains (a viable microbial dietary supplement that beneficially affects the host through its effects in the intestinal tract) but not by other intestinal bacteria [2]. The most studied carbohydrates in the light of their prebiotic properties are fructooligosaccharides (FOS) and galactooligosaccharides (GOS) [3,4,5]. The inulin-like fructans of large and short chain (lcFOS and scFOS, respectively) have been widely used in infants and the evidence leads a considerable safety and bifidogenic effect [6,7]. At the same time, infant formulae are increasingly being supplemented with probiotics, prebiotics, or symbiotics despite uncertainties regarding their efficacy [8,9,10].

Concerning to the source of prebiotics, Mexico is considered both the origin of and the biodiversity center for the Agavaceae family, with 117 of the 155 species (75%) belonging to the genus *Agave* native to this country [11]. *Agave tequilana* Weber cv. Azul is the most widely cultivated species. This Mexican Agave plant (*tequilana weber* Blue Variety) is an interesting source of fructans, which are formed by a complex mix of FOS with prebiotic actions [12,13]. Indeed, it had been demonstrated the bifidogenic and physiologic effects of these fructans *in vitro* and in animal models [13,14].

Metlin**^®^** and Metlos**^®^** are the fructans obtained from *A. tequilana* var Weber with β (2→1) and β (2→6) linked fructofuranosyl units, resulting in branched molecules of high solubility and purity. Previously, it was demonstrated in rodent models that these fructans were not toxic, neither at a cellular nor at a genetic level [15]. Added to this, our group demonstrated their safety in newborns when they were incorporated into infant formula [16]. Considering such evidences, we studied the efficacy as prebiotics of Metlin**^®^** and Metlos**^®^**, in term newborn babies from a Randomized Controlled Trial (RCT-NCT 01251783). Our results on changes in gut microbiota, immune response, levels of C-reactive protein, serum ferritin, cholesterol, triglycerides, and lipoproteins, and bone metabolism, are discussed in order to support the efficacy of both fructans as prebiotics.

## 2. Experimental Section

### 2.1. Subjects and Study Design

Biological samples were taken from individuals who participated in a prospective, randomized, controlled, double blind study, with a pilot study design, conducted from February to August 2010. The Research and Ethics Committee of the National Institute of Pediatrics at the Mexican Ministry of Health approved the trial (Registration number 72/2009). All parents signed informed consent for the study. Clinical trials were performed with 600 infants, any gender, all were born at term, adequate for gestational age (birth weight: 2490–3450 g) and aged 20 ± 7 days at recruitment, in apparently good health, without medical history of maternal pathologies during pregnancy. The sample size was calculated, considering the primary outcomes of safety, an α error of 0.05, a β error of 0.10 and a 20% of attrition. Inclusion criteria were (a) the infant was clinically healthy; (b) was term born; (c) age ≤ 27 days; (d) weight ≥ 2490 g; (e) no allergic response to cow milk proteins; and (f) signed written consent. Major exclusion criteria were (a) evidence of hearth, respiratory, gastrointestinal, hematologic or metabolic diseases; (b) mother with a medical story of diabetes (gestational diabetes was accepted if the infant weight at born was ≤ to that of percentile 95); (c) the infant was product of a multiple delivery (twins, triplets, *etc.*).

Sealed envelopes were prepared containing the sequence of treatment assignation, which were obtained using random allocation software version 1.0.1 (Microsoft, Redmond, WA, USA), through a balanced blocking process. Products under research were coded with seven characters printed in the outside of the box. Neither personnel who worked on the study nor parents were informed about the identity of research products. The study personnel did not make any analysis to identify the study products. The principal researcher was assured that, if necessary, the blind would be broken. Infant formula fulfills the nutrient levels for infants according to the regulations written in *The Infant Formula Act 1986*; also contains the two fructans from Mexican Agave “Metlin**^®^** and Metlos**^®^**” (as previously indicated for each group [16]). Parents were instructed to administrate the formula *ad libitum* as the only nutritional source, until the fourth visit. After the fourth visit, there was no restriction about complementary food. In case of acute diarrhea or dehydration, the participants could use rehydration therapy without prebiotics. It was recommended not to use any other drug that could modify the outcomes.

Allocation process classified infants into five groups: Group 1: Formula added with probiotics (*Lactobacillus rhamnosus* was the probiotic used in all the groups) + Metlin**^®^** + Metlos**^®^**; Group 2; Formula added with probiotics + Metlin**^®^**; Group 3: Formula added with probiotics + Metlos**^®^**; Group 4; Formula added with probiotics, and Group 5: Formula without probiotics and prebiotics. A reference group of breast milk feeding was included too.

Babies were clinically evaluated monthly, until they reached three months of age. Previously, we demonstrated the safety of this dual system of fructans by means of the analysis of stools frequency, consistency of stools and gastrointestinal intolerance (frequency of abdominal distension, flatulency, regurgitations, vomiting) [16]. Efficacy outcomes include the changes on gut microbiota, levels of saliva IgA, C-reactive protein and serum ferritin, triglycerides, cholesterol, and lipoproteins, and urine deoxipyridinoline (DPD), along the study.

### 2.2. DNA Purification from Feces

We tried to obtain two samples of feces from every baby in the study, the first at baseline and the second after three months. In order to avoid contamination by parents at the time to handle feces, free chemical diapers were used to collect the fecal samples when they attended to their pediatric appointments at National Institute of Pediatrics, Mexico (previously they were opportunely scheduled). Immediately after samples were collected, they were chilled on ice and processed to isolate DNA with QIAamp DNA stool mini kit (Qiagen) as previously described [17]. DNA samples were eluted in a final volume of 100 μL of water each one.

### 2.3. Gut Bacteria

Real-time PCR was performed over 148 samples representing the baseline and three months after treatment along the study for the five groups of treatment and the reference group (maternal milk feeding). DNA from all fecal samples was subjected to 5′-nuclease (TaqMan) real-time PCR assay with fluorogenic probes for 16S rDNA gene sequences of *Bifidobacterium* spp. [18], Enterobacteriaceae [19], *Lactobacillus* spp. [20]; *Clostridium* Cluster XI [21] and total bacteria [22]. TaqMan assays for *Veillonella* spp. and *Bacteroides fragilis* were designed for us (primers and probes are listed in Table 1).

**Table 1:** Primers and probes used in this study.

Target Organisms (Amplicon Size)	Primer/Probe	Sequence (5′ to 3′)	*T_m_*, °C	Source
*Bifidobacterium* spp. (231 bp)	Forward primer	GGGATGCTGGTGTGGAAGAGA	60	Haarman & Knol, 2005 [18]
	Reverse primer	TGCTCGCGTCCACTATCCAGT	60	
	Probe	VIC-TCAAACCACCACGCGCCA-NFQ-MGB	70	
Enterobacteriaceae (96 bp)	Forward primer	CATGCC GCGTGTATGAAGAA	59	Huijsdens *et al.*, 2002 [19]
	Reverse primer	CGGGTAACGTCAATGAGCAAA	59	
	Probe	6-FAM-TATTAACTTTACTCCCTTCCTCCCCGCTGAA-TAMRA	68	
*Lactobacillus* spp. (92 bp)	Forward primer	TGG ATG CCT TGG CAC TAG GA	58	Haarman & Knol, 2006 [20]
	Reverse primer	AAA TCT CCG GAT CAA AGC TTA CTTAT	58	
	Probe	VIC-TATTAGTTCCGTCCTTCATC-NFQ-MGB	68	
*Clostridium* Cluster XI (139 pb)	Forward primer	ACGCTACTT GAGGAGGA	58	Nakamura *et al.*, 2009 [21]
	Reverse primer	GAGCCG TAG CCT TTC ACT	58	
	Probe	6-FAM-GTGCCAGCAGCCGCGGTAATACG-TAMRA	63	
*Bacteroides fragilis* (99 bp)	Forward primer	CTACAGGCTTAACACATGCAAGTC	54	This study
	Reverse primer	GCAGGTTGGATACGTGTTACTCA	54	
	Probe	6-FAM-TCGCCAGCAAAGAAA-NFQ-MGB	64	
*Veillonella* spp. (128 bp)	Forward primer	ATCAACCTGCCCTTCAGAGG	54	This study
	Reverse primer	AATCCCCTCCTTCAGTGATAGCTTA	54	
	Probe	6-FAM-TAGCAGTCGTTTCCAACTGT-NFQ-MGB	68	
Total Count (467 bp)	Forward primer	TCCTACGGGAGGCAGCAGT	59	Nadkarni *et al.*, 2002 [22]
	Reverse primer	GGACTACCAGGGTATCTAATCCTGTT	58	
	Probe	6-FAM-CGTATTACCGCGGCTGCTGGCAC-BHQ1	70	

*T_m_* indicates melting temperature; bp, base pairs.

We verified the specificity and the optimal primer and probe concentration for the best amplification efficiency of each TaqMan assay with the following representative DNA reference strain isolates: *Bacteroides fragilis* (ATCC 25285D, EN-2), *Escherichia coli* (ATCC 700928D-5, CFT073), *Clostridium difficile* (ATCC BAA-1382D-5, 630), *Shigella flexneri* (ATCC 700930D-5, 2457T), *Bifidobacterium adolescentis* (ATCC 15703D, E194a Variant a), *Bifidobacterium breve* (ATCC 15700D-5, S1 Variant a), *Bifidobacterium infantis* (ATCC 15697D-5, S12), *Lactobacillus casei* (ATCC 334D-5, ATCC 334™), *Lactobacillus acidophilus* (ATCC 4357D-5, Pak), *Lactobacillus delbrueckii* subsp. *bulgaricus* (ATCC 11842D-5, Lb14), *Veillonella parvula* (ATCC 10790D-5, Te3) and *Enterococcus faecalis* (ATCC 700802D-5, V583). Standard curves for each species were constructed within a dynamic range (0.32 to 1,000 pg of reference bacterial genomic DNA) for relative quantification purposes.

All reactions were carried out by triplicate in a total volume of 25 μL, containing 1X TaqMan Universal PCR Master Mix with uracil-DNA glycosylase (Applied Biosystems, Foster city, CA), 500 nM/L of both primers and 250 nM of each TaqMan probe and 500 pg of purified fecal DNA. The amplification (2 min at 50 °C, 95 °C for 10 min and 40 cycles of 95 °C 20 s. and 1 minute at 60 °C), the fluorescence detection, and analysis were conducted in an Applied Biosystems 7000 Real-time PCR System (Applied Biosystems). Investigators who conducted the measurement were blinded.

### 2.4. Saliva IgA

Two samples of saliva were collected for every child who participated in the study. One sample was collected at baseline, and a second sample was taken at the end of the study (when each baby was three months of age). Plastic sterilized pipettes were used to collect saliva samples and they were immediately stored in 0.25 mL microtubes at −20 °C until they were analyzed (no more than a week after). IgA concentration in saliva was calculated by ELISA (enzyme-linked immunosorbent) assay using anti-IgA antibody coupled to peroxidase. Assays were performed in plastic ELISA plaques and measured in a spectrophotometer at 490 nm. Investigators who conducted the measurement were blinded.

### 2.5. C-Reactive Protein and Serum Ferritin, Triglycerides, Cholesterol, and Lipoproteins

When authorized (authorization to take blood sample was solicited to parents albeit they previously had signed the informed consent), approximately 3 mL of venous blood were obtained by venipuncture procedure in the arm. The whole blood was clotted at room temperature approximately for 15–30 min. The clot was removed by centrifugation and the resulting supernatant, was carefully removed using a Pasteur pipette and stored at −20 °C in microtubes until analysis. Two samples were taken, one at baseline and a second one at the end of the study. Cholesterol and triglycerides were quantified by enzymatic methods in the auto analyzer Express 550, Ciba brand Corning with reference methods of the Center for Prevention and Disease Control, (Atlanta GA Center for Disease Control) (19829 S-N 631-008-24542 CDC). To measure high-density lipoprotein (HDL-C), those whose density was less than 1063 were precipitated, and the HDL-C in the supernatant were analyzed by the same enzymatic method. Total lipids were measured by using conventional colorimetric methods available in the market (Merck). Low-density lipoproteins (LDL-C) and very low-density lipoproteins (VLDL-C) were estimated according to the Friedewald equation [23]:

LDL-C = *Total Cholesterol* − (HDL-C + VDL-C) (1)

VLDL-C = *Triglycerides*/5 (2)

### 2.6. Bone Metabolism

Two urine samples were collected, one sample was collected at baseline, and a second sample was taken at three months of age for each baby. Free chemical diapers with a sterilized urine collecting bags were used to collect the urine samples at the time of the pediatric appointment at National Institute of Pediatrics (previously they were opportunely scheduled). Samples were frozen at −70 °C until were processed. In order to evaluate bone resorption, excreted deoxipyridinoline (DPD) was measured using enzyme immunoassay (MicroVue DPD EIA kit, Quidel Corporation, San Diego, CA, USA). Due to the difficulties inherent to the recollection of this kind of samples, we analyzed only 264 samples. Investigators who conducted the measurement were blinded.

### 2.7. Statistical Analyses

Statistical analyses were performed by the use of IBM SPSS Statistics Version 20.0 software (IBM Corporation, Armonk, NY, USA). To ensure the comparability of the groups the numerical variables were contrasted by means of ANOVA (Analysis of Variance) or its no parametric equivalent, Kruskall Wallis Analysis. If it was considered convenient, a covariant adjustment with Analysis of Covariance (ANCOVA) was performed. When needed, Wilcoxon test and Fisher Student T-Test were applied. A *p* ≤ 0.05 value was used as significant. In all the hypothesis tests, a *p* value less than 0.05 as significant, was used.

## 3. Results

From a total of 937 potentially eligible babies, 187 (20%) had at least one criteria of exclusion. In 150, their parents did not accept to participate in the study. Thus, the final sample size for the study was 600 babies. There were no significant differences among groups in relation with age at the moment of entry in the study, distribution by gender, gestational age, suffocation birth history (APGR), or the birth weight and height, as we previously reported [16].

### 3.1. Feeding Frequency

The reported frequency of breastfeeding in the group of exclusive breastfeed was 9.7 ± 2.7 times in 24 h, meanwhile the group fed with infant formula (groups 1 to 5) received in average 8.3 ± 2.2 intakes in 24 h. When comparing the average quantities of the formula taken for each group during the study (in a range of 639 to 861 mL/day at 1–2 months of age, and in a range of 1076 to 1352 mL/day at three months of age), no differences were found, neither clinically nor statistically significant [16].

### 3.2. Changes in Gut Microbiota

**Figure 1:**
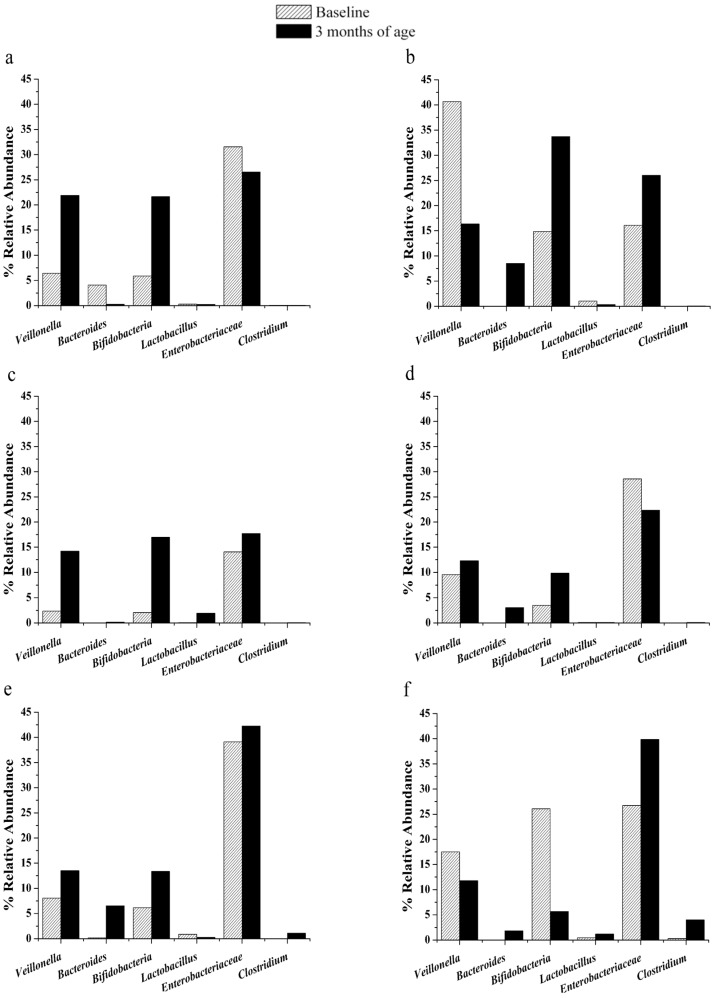
Changes in the relative abundance of gut bacteria, according to the kind of feeding after three months of treatment. Data from total counts were taken as 100% to normalize all the other counts. Reference group of breast milk fed (**a**), and Groups 1, 2, 3, 4, and 5, (**b**–**f**), respectively.

Due to the difficulties on collecting the fecal samples (this was at the time of the pediatric appointment), we were able to collect only 600 samples of feces; 480 samples were of enough quality to isolate DNA and only 300 were from patients who completed the first and the second sampling. From the latter, 148 samples were subjected to real-time PCR. Figure 1 depicts the changes observed among six different groups of bacteria from the data at baseline and three months later for all the groups of study along the clinical trial.

The groups of bacteria analyzed here are those whose role in the intestinal health has been frequently mentioned as relevant for humans. On this, the bacterial groups of *Veillonella,* Enterobacteriaceae, *Clostridium* and the Bacteroidetes are commonly more abundant in infants fed with formula than with breast milk [24]. On the other, the genera *Bifidobacterium* and *Lactobacillus* are examples of bacteria in the colon that have the potential to improve the health of the host and it has been reported an increase of such bacteria in breastfed children [25,26,27].

Our results show a very similar abundance of the genus *Veillonella* in all the groups regardless of the kind of feeding. Abundance of the genus *Bacteroides* was low and variable among groups but only the breastfed group showed a decrease at the time of second sampling. Enterobacteriaceae was abundant in all the groups regardless of the kind of feeding; nonetheless, the groups of breastfed (Figure 1a) and formula + Probiotic + Metlos (Figure 1d) showed a slight decrease. The others showed increase on Enterobacteriaceae from the lowest to the highest; formula + Probiotic (Figure 1e) < formula + Probiotic + Metlin (Figure 1c) < formula (Figure 1f) < formula + Probiotic + Metlin + Metlos (Figure 1b), respectively.

Interestingly, the changes on abundance of Bifidobacteria showed a tendency to increase in those groups fed with breast milk and with fructans and probiotics. Therefore, a bifidogenic gradient was identified, from the highest to the null (times that abundance changed between samples from three months of age and baseline), as follows: formula + Probiotic + Metlin (8.24 times) (Figure 1c) > Breast milk (3.69 times) (Figure 1a) > formula + Probiotic + Metlos (2.8 times) (Figure 1d) > formula + Probiotic + Metlin + Metlos (2.27 times) (Figure 1b) > formula + Probiotic (2.18 times) (Figure 1e) > formula (−4.86 times) (Figure 1f).

*Lactobacillus* population was very low in all the studied groups (no more than 1.3%) and there was no correlation with the kind of feeding.

**Figure 2:**
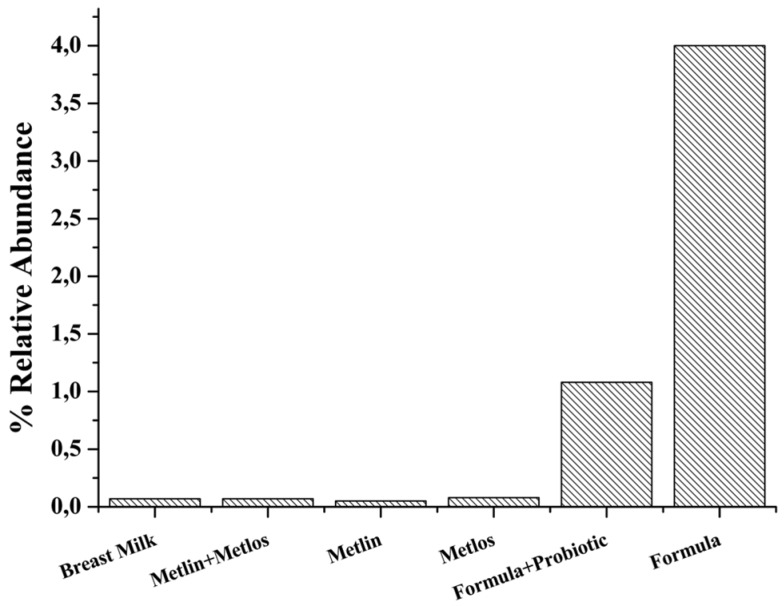
*Clostridium* population changes after three months of age in the different groups of study.

Although *Clostridium* showed low quantities in all the groups, the most evident differences were between those groups fed with breast milk or formula added with fructans than those fed with formula without fructans (Figure 2). In fact, *Clostridium* population in those babies fed with formula alone was 57 times higher than those of reference group or groups containing fructans in the formula. In the group fed with formula + Probiotic, *Clostridium* population was 15 times higher than in the others. Between reference group (breast milk feeding) and groups fed with any of the tested fructans, the relative abundance of *Clostridium* was almost the same.

Results did not change substantially when data from vaginal birth and cesarean section were analyzed independently.

### 3.3. Immune Response

An exploratory analysis for immunity was made through the IgA saliva levels, identifying differences with statistical significance between the group of infants fed with breast milk and those fed with formula enriched with the combination of Metlin^®^ and Metlos^®^ (Group 1), when comparing the values at the end of the monitoring with the value obtained at baseline (Table 2). The grade of immune system stimulation according to the kind of feeding was from the major to the lowest as follows: formula + Probiotic + Metlin + Metlos > formula + Probiotic + Metlin > formula + Probiotic + Metlos > Breast milk > formula + Probiotic > formula.

**Table 2:** Comparative analysis of the saliva immunoglobulin A levels.

Variable	Probiotics + Metlin + Metlos (*n* = 93)	Probiotics + Metlin (*n* = 93)	Probiotics + Metlos (*n* = 89)	Probiotics (*n* = 89)	Only Formula (*n* = 89)	Breast Milk (*n* = 147)
IgA in Saliva Basal (μg/mL) (*X* ± SD)	11.8 ± 8.5	14.1 ± 10.1	12.1 ± 8.3	11.6 ± 7.7	14.0 ± 9.9	13.9 ± 9.3
IgA in Saliva Final (μg/mL) (*X* ± SD)	26.6 ± 11.8	24.8 ± 13.3	23.1 ± 11.4	21.3 ± 8.9	21.7 ± 9.7	24.4 ± 11.4
Differences IgA in Saliva (μg/mL) (*X* ± SD)	17.7 ± 8.1 **	13.9 ± 6.5	12.6 ± 7.5	11.1 ± 10.5	9.6 ± 6.1	12.3 ± 8.3

* *p* < 0.05; ** *p* < 0.01; If not specified, the differences were not statistically significant.

### 3.4. C-Reactive Protein, Serum Ferritin, Cholesterol, Triglycerides, and Lipoproteins

Comparing the average of the mean concentration of samples at the end (three months of age), those at baseline (around 20 days old), and the variables ΔC-reactive protein concentration and Δserum ferritin, the differences were not statistically significant (Wilcoxon test *p* = 0.05) among groups (Table 3). When data were analyzed by comparison of cesarean and vaginal delivery, the differences were not statistically significant in relation to the concentrations of C-reactive protein (initial, final) and ferritin (initial, final) (Fisher Student *T*-Test: *p* > 0.05).

**Table 3:** Levels of C-reactive protein and serum ferritin.

Group	*n*	C-Protein Initial (mg/dL) Median Mean 95%CI	C-Protein Final (mg/dL) Median Mean 95%CI	Ferritin Initial (ng/mL) Median Mean 95%CI	Ferritin Final (ng/mL) Median Mean 95%CI
Probiotics + Metlin + Metlos	27	0.290		328.0	146.0
		0.329		382.74	155.26
		0.248 to 0.410		318.1 to 447.2	113.3 to 197.2
Probiotics + Metlin	32		0.290	348.5	147.5
			0.296	379.46	161.0
			0.282 to 0.310	333.4 to 425.4	121.0 to 200.9
Probiotics + Metlos	25		0.290	264	129.0
			0.295	281.96	131.05
			0.287 to 0.302	244.5 to 319.3	97.4 to 167.7
Probiotics	31	0.290	0.290	331.0	125
		0.297	0.402	337.28	135.78
		0.285 to 0.308	0.173 to 0.631	283.1 to 391.4	98.31 to 173.2
Only formula	20		0.290	321	137.5
			0.291	330.65	153.43
			0.288 to 0.294	262.3 to 398.9	111.3 to 195.5
Breast Milk	56		0.290	360.0	134.0
				348.11	154.76
			0.374 to 0.484	306.1 to 390.0	126.9 to 182.6

ANOVA, Analysis of Covariance, *p* > 0.05.

It was evident that serum concentrations of total cholesterol, triglycerides and lipoproteins were significant different among the groups at the end of the study, with significantly lower values in the group supplemented with Metlin + Metlos + probiotics (Table 4).

**Table 4:** Levels of triglycerides and cholesterol.

Parameter	Group Probiotics + Metlin + Metlos (*n* = 93)	Group Probiotics + Metlin (*n* = 93)	Group Probiotics + Metlos (*n* = 89)	Group Probiotics (*n* = 89)	Group only Formula (*n* = 89)	Group Breast Milk (*n* = 147)
Triglycerides (mg/Dl)	121 ± 8 *	124 ± 23	126 ± 40	125 ± 18	142 ± 30	123 ± 11
Total Cholesterol (mg/Dl)	123 ± 6	125 ± 13	124 ± 17	124 ± 8	132 ± 11	120 ± 19
HDL-Cholesterol	48 ± 7 *	41 ± 7	43 ± 11	42 ± 10	32 ± 6	49 ± 11
LDL-Cholesterol	38 ± 7 *	38 ± 8	40 ± 6	41 ± 16	65 ± 14	36 ± 11
VLDL-Cholesterol	25 ± 11	27 ± 14	27 ± 8	26 ± 17	28 ± 4	23 ± 11

* *p* < 0.05; ** *p* < 0.01; If not specified, the differences were not statistically significant.

### 3.5. Bone Metabolism

DPD concentration in urine was calculated on samples from the first visit (admission) and in the last visit (three months later); average values of the difference between those concentrations were calculated. The recorded values were as follows: Reference group (breast milk feeding): 1020 nmol/L. Group 1: 1100 nmol/L; Group 2: 1015 nmol/L; Group 3: 900 nmol/L; Group 4: 750 nmol/L; Group 5: 600 nmol/L. It can be observed a tendency of DPD to increase from those groups fed without fructans to those groups fed with Metlin**^®^** and/or Metlos**^®^** till reference group with the highest levels of DPD.

## 4. Discussion

The equilibrium of the intestinal human ecosystem is very important in the development of each individual and is composed of anaerobic and aerobes bacteria, yeast, and fungi. Factors that control such equilibrium of microbial populations in the intestine are vast and interact in complex ways, which are not fully understood until today. One of the essentials on this process is the colonization and establishment of microbiota. The first step begins at birth (phase 1 colonization), just at the moment when the product leaves the intrauterine environment. After that, newborns continue their gut colonization during about six months (phase 2 of colonization) until weaning (phase 3 of colonization). Until about 18 months of age, the intestinal microbiota is completed, which includes around 500–1000 different species, mostly of bacteria.

Resident microbiota influences in a good or bad manner the balance of the intestinal ecosystem. When the variety and quantity of the intestinal microbiota propitiates the prevalence of beneficial bacteria, it can generate a long-term protection against pathogenic bacteria (colonization resistant); as an example, *Lactobacillus* and *Bifidobacterium* are considered health promoters. Taking into account that microbiota composition is influenced by the kind of initial oral alimentation; therefore it is reported that in the breastfed infants predominance of *Bifidobacterium* and *Lactobacillus* exists, meanwhile in infants fed with formula Enterobacteriaceae, *Clostridium,* and the Bacteroidetes predominate [24].

Nowadays, nutritional strategies offer an option to influence the balance of the intestinal ecosystem with the aim of favor the establishment of bacteria species that promote conditions of good health in the host; one of these strategies is the addition of prebiotics to food.

Some works have shown a bifidogenic effect using infant formulae added with prebiotics [18,28,29]. Fructans from Agave plant have been suggested as potential prebiotics [16]. On this way, it is important to note that our results show an increase of relative abundance on bifidobacterial population in all groups, except for that fed with formula alone (Figure 1). A bifidogenic effect, characteristic of some prebiotics, is clearly evident in the group fed with the mix of formula + prebiotics + Metlin in contrast with a decrease of bifidobacterial population in the group fed only with formula (Figure 1c *vs.* Figure 1f). In contrast to the evident bifidogenic effect, *Lactobacillus* abundance was very low despite of the addition of *L. rhamnosus* in the infantile formula. Such results can be explained based on the specificity of the primers we used; it means that *L. rhamnosus* could be not detected [20] and, consequently we could not follow the changes of these bacteria in the groups of study. Nonetheless, neither in the reference group (breastfed) nor in the other groups could be established a tendency (related with the kind of feeding) to increase the *Lactobacillus* species detected by the primers used on this work. Indeed, it has been suggested that lactobacilli are unable to form stable populations in the infant [30]. The specialized literature continuously suggests that the establishment of a “beneficial microbiota” (*i.e.*, probiotic microbiota) influences the colonization by other groups of bacteria. The variability of our results on relative abundance of *Veillonella*, *Bacteroides*, and Enterobacteriaceae did not allow us to find any correlation with the observed bifidogenic effect. Nonetheless, the distribution of *Clostridium* populations among the groups of study showed a clear tendency to decrease in those individuals fed with breast milk or with formula added with fructans and probiotics. It correlates with the majority of previous works using prebiotics and probiotics.

Other benefits commonly attributed to the supplementation with a different mix of fructans are an antiallergic effect in the pediatric stage and low incidence in atopic dermatitis [31,32]; moreover, significant qualitative and quantitative differences in the bifidobacterial microbiota composition of allergic and healthy children have been reported [33,34]. IgA level is one of the first responses in the human host when the immune system is stimulated or depleted [35]. Our results on IgA levels in saliva suggest an activation of the immune system in those groups fed with formula added with both or any of the used fructans, and the lowest immune response in those groups fed without fructans (Table 2). These results show a tendency to stimulate the immune system using fructans in formula and underline again the poor effect in the group fed with formula alone.

According to the results of C-reactive protein and serum ferritin, they denote that children in the study did not show processes of inflammation related to infection; neither at the moment of recruitment nor after the time they were in the study, and that the kind of delivery has no influence on the concentration of C-reactive protein and serum ferritin. Iron is an essential nutrient and plays a key role in many processes including growth and development. Iron deficiency in infancy is associated with a range of clinical and developmentally important issues [36]. The levels of serum ferritin determined in this work indicate availability of iron stores as in other groups of healthy individuals.

Modulation of the lipid metabolism either in its digestion or absorption is another physiological effect attributed to the fructans [37]. Evidence from the clinical studies in healthy adults and preschool supplemented with fructans show reduction of triglyceridemia and decrease in the cholesterol concentration [38]. Our results show a clear tendency to reduce high levels of triglycerides and cholesterol in groups consuming fructans. Statistical significance of the levels of triglycerides, HDL, and LDL in the group fed with the mix of formula + probiotic + Metlin + Metlos is to take into account as one of the more impacting effects of these fructans in the groups of children studied here (see Table 4).

With respect to the bone metabolism, it was previously demonstrated in animal models that intake of Agave Fructans could provide the prevention of bone mass loss and bone weakening, what in turn could improve quality of life, by helping prevent osteoporosis [14]. Although no significance was found, a tendency on increase of DPD in groups fed with fructans and breast milk was observed. DPD is a bone resorption marker that in children is released during the process of bone growth [39]. Our results show a tendency to an increase of DPD in infants breast-fed and those with a formula added with fructans. It could be interpreted as a high bone turnover related to fructans and breast milk consumption; nonetheless, DPD and other markers analyzed in urine are influenced by other variables that we cannot control (circadian cycle, levels of creatinine, *etc.*)

## 5. Conclusions

Finally, according to Roberfroid’s definition of prebiotic (A prebiotic is a selectively fermented ingredient that allows specific changes, both in the composition and/or activity in the gastrointestinal microbiota that confers benefits upon host well-being and health), the fructans from Mexican Agave (Metlin^®^ and Metlos^®^) promote a tendency of changes of microbiota (bifidogenic effect) and benefit the host in the sense of activating immune system and regulating triglycerides, cholesterol, and lipoproteins levels; therefore, our results are very suggestive to propose the fructans from Mexican Agave (Metlin^®^ and Metlos^®^) as efficient prebiotics for newborns, and these data will help us to design a more definitive study.
